# Outcomes of Patients Transferred to Tertiary Center by Life-Saving System in Saudi Arabia. A Propensity Score Matching Observational Study

**DOI:** 10.2478/jccm-2024-0038

**Published:** 2024-10-31

**Authors:** Mohammed Soliman, Hanan Alenzi, Rehab Alfenaikh, Ahmed Aletreby, Malak Alenzi, Hend Alenzi, Jennifer Gano, Rana Alrashed, Yasmeen Altaymani, Mohammed Al-Odat, Waleed Aletreby

**Affiliations:** King Saud Medical City, Riyadh, Ar Riyad, Saudi Arabia; Faculty of Medicine, Alexandria University, Alexandria, Egypt; Imam Abdulrahman Alfaisal Hospital, Riyad, Saudi Arabia

**Keywords:** intensive care, inter-hospital, transfer, length of stay, life-saving, mortality

## Abstract

**Background:**

Inter-hospital transfer is intended to provide access to centralized special care for critically ill patients, when resources in their hospitals are not available. However, an empirical gap exists in available evidence, as outcomes of transferred patients to higher centers are inconsistent.

**Method:**

Single center propensity score matching retrospective observational study. Life-Saving transfers during 2023 were matched to direct admissions to the ICU. Hospital mortality, ICU length of stay, and costs of both groups were compared.

**Results:**

During the study period, 328 Life-Saving transfers were matched to 656 direct admissions. Propensity score matching eliminated all imbalances between groups. Hospital mortality was not different between groups, there were 114 (34.8%) hospital mortalities of Life-Saving transfers, while there were 216 (32.9%) hospital mortalities of direct admissions, with a percent difference of 1.9% (95% CI: −4.5%, 8.4%); p value = 0.6, this result persisted in the sensitivity analysis. There were no differences in mortality risks for all the studied subgroups except pediatric patients. ICU length of stay of direct admissions and Life-Saving transfers were 10 ± 13.1 and 11.6 ± 12.4 days respectively, mean difference was statistically significant (−1.6 [95% CI: −3.2, 0.1]; p = 0.005). Life-Saving transfers entailed significantly higher costs per admission by 28,200 thousand SAR (95% CI: 26,400 – 30,000; p < 0.001).

**Conclusion:**

Our study shows no difference in hospital mortality between Life-Saving transfers and direct admissions to ICU, however, Life-Saving transfers had a longer ICU length of stay, and higher costs per admission.

## Introduction

Inter-hospital transfer (IHT) systems are intended to facilitate access to care for critically ill patients, when the required services or resources in the primary hospital are lacking [1]. Ideally, IHT would be most beneficial when the transfer decision is solely based upon the patients’ medical needs, and is not influenced by any other considerations, such as social reasons or family preferences [2].

If implemented in such a manner, IHT systems would be expected to result in better patients’ outcomes, since they - presumably – receive high quality care, and exploit available expertise and resources in the receiving hospital [3]. However, evidence from studies are at conflict, with several studies reporting worse outcomes for transferred patients as compared to those directly admitted to intensive care units (ICUs), such as higher mortality rates [1, 4, 5], longer ICU or hospital length of stay (LOS) [1, 5,6,7]. Even considering the body of evidence indicating similar outcomes [3, 8, 9], IHT is undoubtedly costly, and inherently carries the risks of adverse events during the transfer process itself, owing to several reasons like the critical condition of the transferred patients themselves, the distance and duration of transfer, and the level of care provided during transfer [2, 8, 10]. Notably, IHT usually takes place for trauma cases, whether blunt or penetrating, cases of sepsis and septic shock, cardiac conditions such as cardiac arrest or infarction, in addition to respiratory conditions like infections and COPD [8, 9].

The Ministry of Health (MOH) in Saudi Arabia utilizes a system of Life-Saving transfer that is intended to avoid delays in access to care or services for critically ill patients with life threatening conditions, or at risk of an organ or a member loss. The treating consultant of such cases in the primary or secondary hospitals contacts the unified Life-Saving hot-line to request an urgent transfer to a tertiary referral hospital. Approval of transfer is granted by the MOH and is not required from the receiving hospital, once the transfer is approved, arrangement for transfer are made by the patients’ original hospital, and the receiving hospital is only informed about the transfer and the patients’ condition. Upon arrival, they land in the emergency department (ED), where the ICU and the specialty to which they were referred are informed to start their management [11].

In view of the conflicting evidence of the outcomes of IHT and the scarcity of such studies in our population, we conducted this study with the aim of comparing outcomes of patients transferred by the Life-Saving system and patients directly admitted to ICU from the ED or general wards. We hypothesized that Life-Saving transferred patients may have worse outcomes.

## Materials and Method

This was a retrospective observational study, performed in the ICU of the largest tertiary referral hospital in the central region of Saudi Arabia. The ICU harbors 125 beds, fully equipped with invasive and non-invasive monitoring and ventilation capabilities. It is a closed ICU, operated round the clock by intensivists, and has a nurse to patient ratio of 1: 1. Although our ICU is an adult ICU, we still sometimes admit pediatric patients, particularly surgical and trauma cases because of unavailability of those services in the pediatrics hospital.

### Groups’ description

Patients admitted to our ICU through the Life-Saving transfer system from other primary and secondary hospitals of the region comprised the (LS) group, while the direct admission (DA) group included all patients admitted directly to ICU from the hospital’s ED or general wards.

### Study outcomes

The primary outcome was all cause hospital mortality of the LS group compared to the DA group. Secondary outcomes included comparison of ICU LOS between groups, and cost-effectiveness group comparison. As a sensitivity analysis of the primary outcome, we evaluated the association of Life-Saving transfer with mortality controlling for other variables.

Additionally, we performed sub-group analysis of mortality for sex, median age of the whole cohort, median predicted mortality rate (PMR) of the whole cohort, adulthood, and general admission category.

### Timeframe and data management

This study was conducted between January 1, 2023 and December 31, 2023. We reviewed the electronic medical records (EMR) of all patients who were discharged from the ICU during the study period, eligible patients were grouped into LS group or DA groups according to their source of admission. Furthermore, we noted age, sex, PMR calculated from APACHE 4 in the first 24 hours of ICU admission, the general admission category as: Fast-track, Maternity, Medical, Surgical, or Trauma. Fast-track patients are the patients who had a scheduled or emergency major surgery, and required a short period of post-operative ICU monitoring and observation. We also recorded the hospital outcome of each patient as a binary Dead / Alive variable. Notably, patients who were discharged to other healthcare facilities were considered alive, since we could not follow them up. Finally, we calculated the ICU length of stay for each patient.

Outcome data, sex, admission category, and being life-saving or not are certain to be available in the EMR, however, PMR, and age (for unknown patients) may be missing. Records with missing data were not included in the study’s cohort.

### Inclusion and exclusion criteria

We included all ICU patients with a known hospital outcome during the study period, regardless of age, sex, or diagnosis. For patients who had more than one episode of ICU admission within the same hospitalization, we accounted only for the first episode to maintain independency of data, and because the initial admission is probably the one most related to the original patient’s condition. However, hospital outcome was registered at the end of the total hospitalization period. Patients still admitted to the ICU or hospital at the end of the study period (hospital outcome is not known) were not enrolled.

### Minimum required sample

Although this was an observational study, in which we enrolled all eligible patients during the study period, we estimated that if 300 patients in the LS group (based on historical data from our ICU) were matched to 600 patients in the DA group, with an absolute mortality difference of 10%, this would be sufficient to provide a power of 90% at a type I error rate of 5%.

### Statistical method

We summarized continuous data as mean and standard deviation, and discrete data as frequency and percentage. The distribution of continuous data was assessed using Shapiro-Wilk test, and were compared between groups using student t-test if the normality assumption was satisfied, otherwise, the non-parametric Wilcoxon Rank SUM test was used to compare groups. Discrete data were compared using Pearson’s Chi square test, or Fishers exact test as appropriate for the cell counts in the 2 × 2 contingency table. Groups’ comparisons were presented as mean or percent difference, with corresponding 95% confidence interval (CI).

We performed propensity score matching to match each patient in the LS group to two patients in the DA group based on age, sex, admission category, and PMR. The propensity score matching algorithm used “Nearest Neighbor” method (Figure S1), and we assessed the mean-bias reduction of the matching statistically and graphically.

As a sensitivity analysis of the mortality outcome, we fitted a logistic regression model using “enter” method, the model included age, sex, PMR, admission category, and the variable of interest (Group) as independent variables, and hospital mortality as a dependent variable. Logistic regression results were presented as odds ratio (OR) with corresponding 95% CI.

Sub-groups analysis included sex, adulthood, median age, median PMR, and admission category. Subgroups comparisons were presented as risk difference.

All statistical tests were two tailed, and were considered statistically significant if the p values were < 0.05, without correction for multiple testing. Accordingly, results of secondary comparisons should be interpreted cautiously. Freely available statistical software R-studio [12] and the package “MatchIt” [13] were used to perform the statistical analysis.

### Ethical considerations

The study was approved by the local institutional review board with waiver of consent in view of its retrospective observational design. Results are anonymized without reporting of any patients’ identifiers. The study follows the general principles of research subjects’ protection outlined by the declaration of Helsinki. The manuscript was prepared according to the Strengthening the Reporting of Observational Studies in Epidemiology (STROBE) guidelines [14].

## Results

**Fig. 1.**
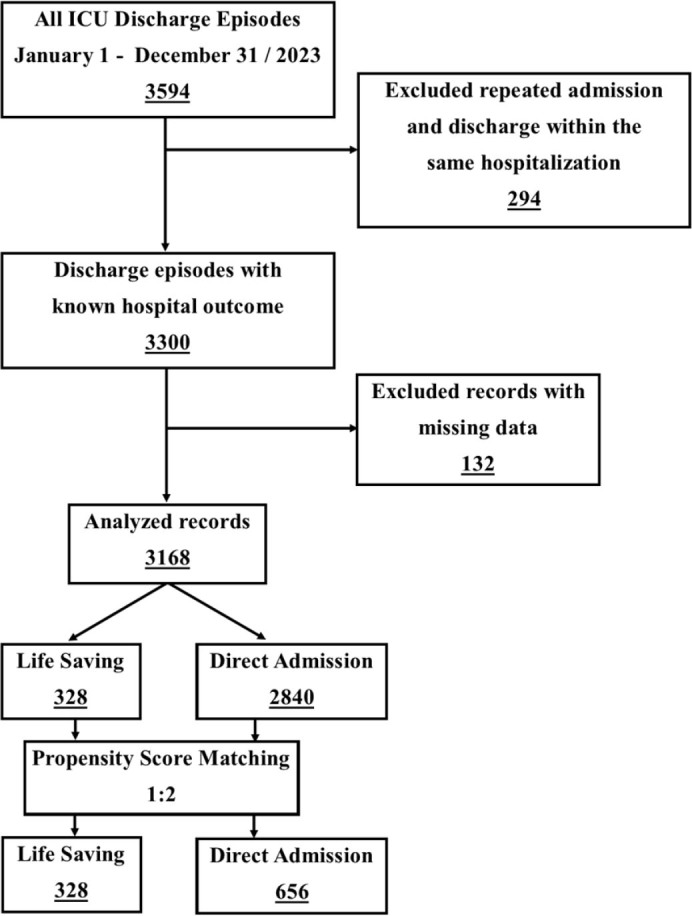
Patients’ enrollment diagram

During the study period, there were 3594 discharge episodes from the ICU. After exclusion of repeated admission and discharge episodes and cases with missing data, we analyzed 3168 records, which included 328 Life-Saving transfers (LS group). All members of LS group were successfully matched 1:2 to available records of direct admissions to generate the DA group (n=656) (Figure 1).

Before propensity score matching, LS and DA groups showed imbalances in sex distribution, categories of admission (except for surgical), and PMR (Table 1). However, propensity score matched groups were totally balanced with regards to all variables, with a 92% mean-bias reduction with successful matching of all LS group members to two DA group members (Figures S2 and S3).

**Table 1.** Unmatched groups’ demographic and clinical characteristics

**Variable**	**DA (n = 2840)**	**LS (n=328)**	**Mean / Percent difference (95% CI)**	**p value**
Age §	45.1 ± 19.4	46.4 ± 20.9	−1.3 (−3.7, 1.05)	0.2*

Sex: Female ‡	1135 (40%)	109 (33.2%)	6.7% (1.1%, 12.1%)	0.02

Admission Category ‡				
Fast Track	268 (9.44%)	6 (1.83%)	7.6% (5.3%, 9.2%)	<0.001
Maternity	430 (15.14)	14 (4.27%)	10.9% (7.8%, 13.2%)	<0.001
Medical	1096 (38.59%)	158 (48.17%)	9.6% (3.8%, 15.4%)	0.001
Surgical	778 (27.39)	93 (28.35%)	1% (−4.1%, 6.4%)	0.8
Trauma	268 (9.44%)	57 (17.38%)	7.9% (3.8%, 12.6%)	<0.001
PMR §	19.1 ± 17.9	23.2 ± 21.3	−4.1 (−6.5,−1.7)	0.004*

DA = Direct admission, LS = Life-Saving, PMR = predicted mortality rate, SD = standard deviation, CI = confidence interval.

* Wilcoxon Rank SUM test.

§ Data presented as mean ± standard deviation,

‡ Data presented as number (percent).

Matched groups had a mean age of 46.4 ± 20.9 and 45.7 ± 20.1 years for the LS and DA groups respectively. In both groups, about one third were females, and about half of the cases were medical admissions. The LS group had a mean PMR of 23.2 ± 21.3, while the DA group had a mean PMR of 23.9 ± 22 (Table 2). The median age of the cohort was 46 years, and the median PMR was 17, those two values were used in the subgroups analysis.

**Table 2.** Matched groups’ demographic and clinical characteristics

**Variable**	**DA (n = 656)**	**LS (n=328)**	**Mean / Percent difference (95% CI)**	**p value**
Age §	45.7 ± 20.1	46.4 ± 20.9	−0.7 (−3.5, 2.04)	0

Sex: Female ‡	220 (33.5%)	109 (33.2%)	0.3% (−6.2%, 6.6%)	0.97

Admission Category ‡				
Fast Track	15 (2.29%)	6 (1.83%)	0.5% (−1.9%, 2.3%)	0.8
Maternity	25 (3.81%)	14 (4.27%)	0.5% (−2.1%, 3.6%)	0.9
Medical	317 (48.32%)	158 (48.17%)	0.2% (−6.6%, 6.9%)	0.98
Surgical	192 (29.27%)	93 (28.35%)	0.9% (−5.3%, 7%)	0.8
Trauma	107 (16.31%)	57 (17.38%)	1.1% (3.9%, 6.4%)	0.7
PMR §	23.9 ± 22	23.2 ± 21.3	0.7 (−2.2, 3.6)	0.8*

DA = Direct admission, LS = Life-Saving, PMR = predicted mortality rate, SD = standard deviation, CI = confidence interval.

* Wilcoxon Rank SUM test.

§ Data presented as mean ± standard deviation,

‡ Data presented as number (percent).

### Outcomes

Table 3 shows the study’s outcomes. There were 114 mortalities (34.8%) in the LS group, and 216 mortalities (32.9%) in DA group. This slightly higher mortality rate in the LS group did not reach the level of statistical significance (percent difference [95% CI] = 1.9% [−4.5% to 8.4%]; p = 0.6).

**Table 3.** Matched groups’ outcome comparisons

**Variable**	**DA (n=656)**	**LS (n=328)**	**Mean / Percent Difference (95% CI)**	**P value**
Mortality ‡	216 (32.9%)	114 (34.8%)	1.9% (−4.5%, 8.4%)	0.6
LOS §	10 ± 13.1	11.6 ± 12.4	−1.6 (−3.2, 0.1)**	0.005*

DA = Direct admission, LS = Life-Saving, LOS = length of stay, SD = standard deviation, CI = confidence interval.

* Wilcoxon Rank SUM test.

§ Data presented as mean ± standard deviation,

‡ Data presented as number (percent).

** 95% CI is generated using student t-test since Wilcoxon Rank SUM test is a non-parametric test.

The average LOS of the LS group (11.6 ± 12.4) was significantly higher than that of the DA group (10 ± 13.1) when compared using Wilcoxon Rank SUM test due to non-normal distribution of the data (Mean difference [95% CI] = −1.6 [−3.2 to 0.1]; p = 0.005).

The sensitivity analysis of the mortality outcome indicates that controlling for all other variables, Life-Saving transfer was not significantly associated with hospital mortality (OR = 1.12 [95% CI: 0.82 – 1.54]; p = 0.5). On the contrary, age and PMR were significantly associated with increased odds of mortality (Table S1).

**Fig. 2.**
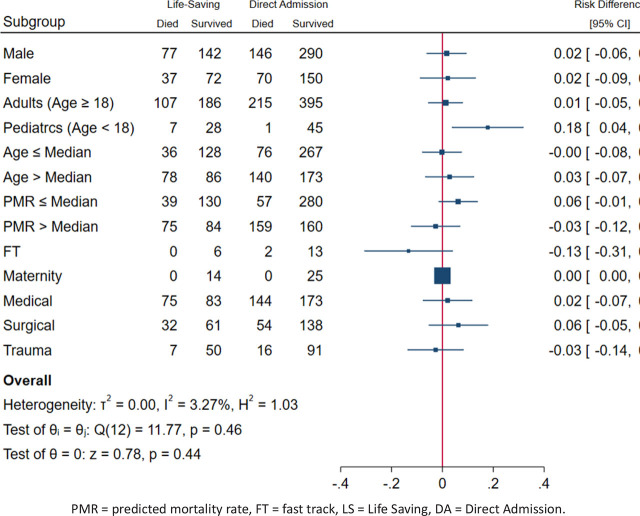
Sub-group analysis

Sub-group analysis indicated that there was no mortality benefit of Life-Saving transfer for any of the explored sub-groups, on the contrary, pediatric patients in LS group had an increased risk of death. There was no evidence of heterogeneity (Figure 2).

### Cost-effectiveness analysis

The LS group accounted for a total of 3810 patient days, while the DA group accounted for 6592 patient days. According to a study previously performed in our ICU [15], the cost of an ICU day can be rounded to 18,000 Saudi Riyals (SAR), equivalent to 4,800 $ at an exchange rate of 3.75. Accordingly, the cost of admission per Life-Saving transfer (calculated as: total inpatient days * cost of a day / number of patients) is estimated to be 209,000 SAR (95% CI: 207,500 – 210,700). While that of the direct admissions is 180,900 (95% CI: 179,900 – 181,900). Life-Saving transfers account for a significantly higher cost of an admission by 28,200 thousands SAR (95% CI: 26,400 – 30,000; p < 0.001).

## Discussion

In this propensity score matching retrospective observational study, mortality rate of transferred patients although numerically higher was not statistically different from that of directly admitted patients, this result was confirmed by the sensitivity analysis controlling for other variables. LOS was higher for transferred patients, who also entailed higher costs. Only the subgroup of pediatric patients had a higher risk of death when transferred by Life-Saving system.

The similar mortality rates of both groups in our study could be the result of the centralized special care the transferred patients received once they landed in our center, and thus their mortality was similar to that of any other patient since we can’t be sure what the mortality rate would have been if they were not transferred. Additionally, the management of transferred patients which has already started in the referring hospital may be a contributing factor [1]. Although this finding of similar mortality rates concurs with several publications [3, 16], yet, it goes counter to many others [5, 17, 18] who report a higher mortality rate of transferred patients. A striking difference between studies with a mortality difference and those without is the sample size. The sample size of positive studies is usually of thousands [4, 6, 18], whereas negative studies tend to have much smaller sample sizes [3, 8, 9]. Indeed our study is among the smaller sized studies, and similarly, it failed to detect a significant mortality difference. Our result of similar mortality rates persisted in the sensitivity analysis after controlling for other variables, indicating that it may be a true finding rather than a result of under-power. Intriguingly, only the subgroup of pediatric patients under the age of 18 years old showed a higher risk of mortality when transferred, this could be the result of adverse events during transfer which can be up to 22% [19], a figure that is very close to the 20% mortality rate of that subgroup in our study, although we can’t be sure that the mortality of transferred pediatric patients is related to adverse events during transport, since those events were not recorded in our study. It remains to be answered whether there is a real mortality difference that underpowered studies fail to detect, or clinically unimportant differences yield statistical significance because of large sample sizes [20].

On the contrary, longer LOS [1, 5,6,7, 21] and higher costs [5, 6, 8] seem to be regular findings in studies addressing the subject, similar to our study. Longer LOS and consequently more costs may be plausible in view of the complexity of transferred patients requiring more laboratory or radiological investigations, the receiving team may have a different perspective and approach to the management of the patient, and don’t always build on what was already started in the original hospital [22].

It is imperative to emphasis that we don’t advocate restricting or limiting IHT, on the contrary, just by having mortality rates of transferred patients similar to that of directly admitted patients, may be enough of a justification for their transfer, since we can’t be sure of their mortality rates should they have remained in an under-resourced hospital. However, we advise regulating and organizing the process, so that patients in real need of specialized care can benefit from the system. Transfer policies may be established, with clear objective criteria of transfer, safe transfer guidelines should be followed, and the transfer process should be closely audited. Further research is called for, since numerous factors related to the transfer process are under studied, such as the time to transfer, duration and mode of transfer, and adverse events during transfer. Any or all of those factors may influence the outcomes of transferred patients. Additionally, exploration of the outcomes of patients who were not transferred to higher centers is in order, to elaborate on the significance of the transfer system.

### Limitations

Our study is subject to several limitations. This was an observational study, and thus suffers all the limitations inherent within the observational design, despite performing propensity matching, which is as close to a randomized trial as can be [23]. This was a single center study, reflecting management in one hospital, so its results may not be extrapolated to other hospitals. Several aspects related to the transfer process – as outlined above – were not measured and so were not adjusted for, so we can’t be sure of their confounding effect on the results. The relatively small sample size may have been insufficient to detect a statistically significant difference in the main outcome. We considered the patients’ general diagnostic category but not the exact diagnosis, and patients’ outcomes may differ in specific diagnoses. Finally, patients who were discharged to other hospitals were considered alive which may undermine the true mortality rate, as their real outcome can’t be confirmed.

## Conclusion

Mortality of Life-Saving transferred patients was similar to directly admitted patients to ICU, transfer was not associated with mortality in the sensitivity analysis, and mortality risk was not different for any of the studied subgroups apart from pediatric patients who were at a higher mortality risk when transferred. Transferred patients had higher LOS and consequently higher costs. Further investigations are required to ascertain benefits of IHT systems, including all variables of the transfer process, and outcomes of un-transferred patients. The transfer system should be organized, monitored, and audited.
